# A positioning QA procedure for 2D/2D (kV/MV) and 3D/3D (CT/CBCT) image matching for radiotherapy patient setup

**DOI:** 10.1120/jacmp.v10i4.2954

**Published:** 2009-10-06

**Authors:** Huaiqun Guan, Rabih Hammoud, Fang‐Fang Yin

**Affiliations:** ^1^ Department of Radiation Oncology Good Samaritan Health System Kearney NE USA; ^2^ Henry Ford Down River Center for Oncology Trenton MI USA; ^3^ Department of Radiation Oncology Duke University Medical Center Durham NC USA

**Keywords:** OBI, CBCT, positioning QA, 2D/2D (kV/MV), 3D/3D (planCT/ CBCT)

## Abstract

A positioning QA procedure for Varian's 2D/2D (kV/MV) and 3D/3D (planCT/CBCT) matching was developed. The procedure was to check: (1) the coincidence of on‐board imager (OBI), portal imager (PI), and cone beam CT (CBCT)'s isocenters (digital graticules) to a linac's isocenter (to a pre‐specified accuracy); (2) that the positioning difference detected by 2D/2D (kV/MV) and 3D/3D(planCT/CBCT) matching can be reliably transferred to couch motion. A cube phantom with a 2 mm metal ball (bb) at the center was used. The bb was used to define the isocenter. Two additional bbs were placed on two phantom surfaces in order to define a spatial location of 1.5 cm anterior, 1.5 cm inferior, and 1.5 cm right from the isocenter. An axial scan of the phantom was acquired from a multislice CT simulator. The phantom was set at the linac's isocenter (lasers); either AP MV/R Lat kV images or CBCT images were taken for 2D/2D or 3D/3D matching, respectively. For 2D/2D, the accuracy of each device's isocenter was obtained by checking the distance between the central bb and the digital graticule. Then the central bb in orthogonal DRRs was manually moved to overlay to the off‐axis bbs in kV/MV images. For 3D/3D, CBCT was first matched to planCT to check the isocenter difference between the two CTs. Manual shifts were then made by moving CBCT such that the point defined by the two off‐axis bbs overlay to the central bb in planCT. (PlanCT can not be moved in the current version of OBI1.4.) The manual shifts were then applied to remotely move the couch. The room laser was used to check the accuracy of the couch movement. For Trilogy (or Ix‐21) linacs, the coincidence of imager and linac's isocenter was better than 1 mm (or 1.5 mm). The couch shift accuracy was better than 2 mm.

PACS number: 87.55.Qr, 87.57.Q‐

## I. INTRODUCTION

On‐board imagers (OBI) using kilovoltage X‐rays (kV) have been installed on linear accelerators (linacs) from multiple vendors. Patient setup and target localization can be improved with the use of OBI, which is critical for intensity‐modulated radiotherapy (IMRT) and radiosurgery (IMRS). The OBI not only allows 2D patient setup, it also acquires cone beam CT (CBCT) for 3D patient setup. The detected differences between the online image (either 2D‐kV image or 3D‐CBCT image) and the patient reference images (either 2D‐DRR images or 3D‐planning CT images) can be automatically transferred to couch motion from the treatment console. However, quality assurance is critical for precise image‐guided radiotherapy (IGRT).^(^ ^1^ ^–^ ^5^ ^)^ For 2D/2D matching, kV/MV allows aggregated acquisition of dual images without rotating the linac gantry. For precision radiation therapy (like IMRS) to sites where bones can be good landmarks, kV/MV images can be adequate for quicker patient setup than kV/kV. In our previously reported OBI QA program,^(6)^ kV/kV was recommended for OBI only, using a cube phantom with two sets of small metal balls or bbs, on either a daily or weekly basis. Here we simplify the procedure by just using one set of bbs for kV/MV matching. Further, planCT/CBCT allows direct 3D/3D tissue‐to‐tissue matching for patient setup. But positioning QA for 3D/3D matching was not reported yet. In this paper, we present a combined QA procedure to check: (1) the coincidence of OBI, PI, and CBCT's isocenters (digital graticules) to linac's isocenter (to a prespecified accuracy); (2) that the positioning difference detected by 2D/2D and 3D/3D matching can be reliably transferred to couch motion.

## II. MATERIALS AND METHODS

**Figure 1:**
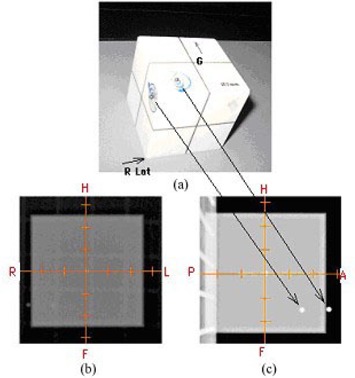
(a) the cube phantom with 2 bbs placed on the surface; (b) the AP MV image; (c) the R Lat kV image.

In our institutions, the OBI and portal imaging (PI) were installed on Varian linacs, either Trilogy or Ix‐21 (Varian Medical Systems, Palo Alto, CA). The Cubic Isocenter Phantom (Fig. 1(a)) provided by Varian was used. The phantom has a 2 mm metal ball (bb) embedded at the center to define the isocenter. We placed two off‐axis bbs on two phantom surfaces in order to define a spatial point 1.5 cm anterior, 1.5 cm inferior, and 1.5 cm right from the isocenter. The off‐axis bb on the anterior surface was placed 1.5 cm right and 1.5 cm inferior from the center, and the off‐axis bb on the right surface was placed 1.5 cm anterior and 1.5 cm inferior from the center. The two bbs shown in the previous study^(6)^ to define isocenter were removed because of the built‐in bb at phantom center. In Fig. 1(a), the gantry direction was labeled as G. An axial scan of the phantom was acquired using a multislice CT simulator with 0.625 mm slice thickness. A plan with three setup fields (AP‐MV, Lat‐kV, and CBCT) was created in Eclipse with digitally reconstructed radiographs (DRR) attached. The difference between the source‐to‐surface distance (SSD, 97.5 cm) to off‐axis bbs and the source‐to‐axis distance (SAD, 100 cm) to the isocenter makes the magnification factor 2.5% larger in kV/MV images than the corresponding DRRs. This may cause 0.25 mm systematic error for couch shift accuracy (based on off‐axis bbs) but not for the isocenter accuracy (based on central‐axis bbs). We generated DRRs with a magnification factor of 1.025 in order to eliminate the error. The 2D/2D QA procedure is as follows:

1) Set up the cube phantom at the linac's isocenter by aligning the anterior crosshair to linac's light‐field crosshair and the lateral crosshairs to lateral lasers
2) Take AP MV and Lat kV images
3) Determine the accuracy of OBI and PI's isocenter by measuring the distance between the central bb and the digital graticule
4) If the accuracy is within specification, manually move the central bbs in DRR images to overlay to the off‐axis bbs in the kV/MV images (If it is not, a service from the vendor is needed.)
5) Document the detected expected shifts and then apply to remotely move the couch
6) After moving, visually check the alignment of room lasers to off‐axis bbs. If not aligned, set the lasers to bbs and record the final couch position. The actual couch shifts are the differences between the initial and the final couch position. The accuracy is the difference between the expected shifts and the actual shifts. Accuracy should be within 2 mm because it is the combination of couch motion accuracy approximately equal to 1.4 mm (1 mm accuracy each before and after the motion) and laser alignment accuracy approximately equal to 1.4 mm (also 1 mm each before and after the motion)

The 3D/3D QA procedure is:

1) Set up the cube phantom at the linac's isocenter
2) Take CBCT image in full fan “High Quality Head” mode using OBI
3) Match planCT to CBCT to check the isocenter difference between the two sets of CTs
4) If that difference is within the specification, manually move the CBCT such that the spatial point defined by the two off‐axis bbs overlay to the central bb in planCT (but not the other way, in the current version of OBI1.4)

The rest of the procedure is the same as above for 2D/2D QA.

## III. RESULTS

**Figure 2:**
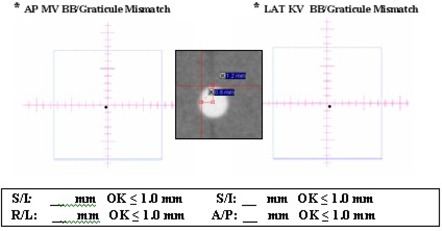
Document of the daily isocenter accuracy in AP (left) and R‐LAT (right) for a Trilogy machine.

**Figure 3:**
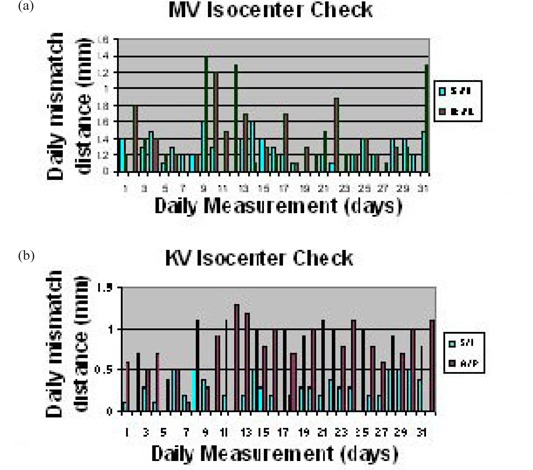
Plots of the isocenter accuracy on (a) AP MV field and (b) R LAT kV field for a Varian Ix‐21 machine over 6 weeks. The means and standard deviations for (a) S/I: 0.31±0.15mm and R/L: 0.49±0.40mm; (b) S/I 0.31±0.13mm and A/P: 0.83±0.27mm.

Figure 1(b) shows the AP MV image and Fig. 1(c) shows the R‐Lat kV image. The two off‐axis bbs on the surface of the cubic phantom were also indicated to their corresponding locations in kV image (Fig. 1(c)). By measuring the distance between the center of central bb (linac isocenter) and the center of digital graticules (imager isocenter) on either the MV or kV image, the accuracy of the OBI or PI's isocenter was obtained, as demonstrated in Fig. 2. The daily document of isocenter accuracy was also shown in Fig. 2 for AP MV (left) and LAT kV (right). Figure 3 represents the six‐week documentation of the isocenter accuracy for a Varian Ix‐21 machine in (a) AP MV (mean and standard deviations: S/I: 0.31±0.15mm and R/L: 0.49±0.40mm), and (b) LAT kV (mean and standard deviations: S/I0.31±0.13mm and A/P:0.83±0.27mm). One sees that the accuracy was better than 1.5 mm. For Troligy, the isocenter accuracy was better than 1 mm.

**Figure 4:**
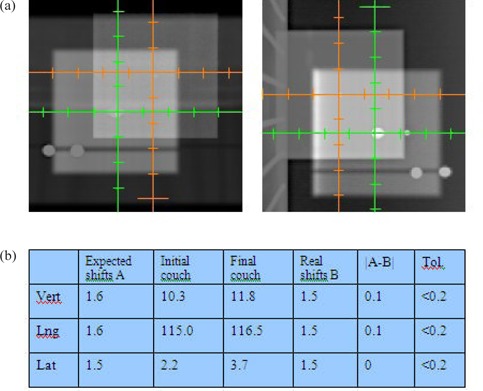
(a) overlay of the central axis bb on DRR to the off‐axis bb on MV (left) and kV(right) images (the shifts will be displayed on the dashboard of OBI); (b) document of couch positions, couch shifts, and tolerances.

If the QA for kV/MV isocenter passes, the central bb in DRR images was manually moved to overlay to off‐axis bbs in kV/MV images (In OBI, 2D/2D manual matching can only be made by moving the DRR image). The matching detected the shifts in three‐dimension, which then applied to remotely move the couch. Figure 4(a) shows the matching of central bb to the off‐axis bbs for MV (left) and kV (right). Figure 4(b) documents the detected (expected) 3D shifts and initial couch position. After the couch movement, the light‐field corsshair (for anterior) and laser (for lateral) alignment to off‐axis bbs were visually checked. If not aligned, the lasers were set to off‐axis bbs and final couch position was recorded. The actual shifts were the difference between the initial and final couch positions. The accuracy of couch shifts (i.e. the |expected ‐ actual|) should be within 2 mm. The final couch position, the actual couch shifts, and the shifting accuracy were also documented in Fig. 4(b).

**Figure 5:**
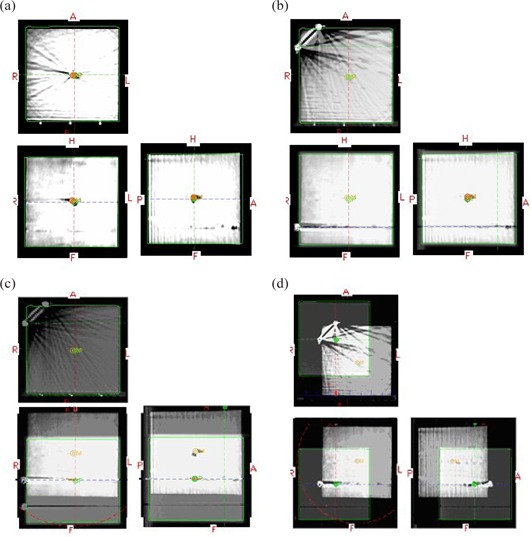
The sequence (a‐d) of moving planCT's isocenter to the off‐axis point, defined by the two off‐axis bbs in CBCT. In each, the top left is the axial view, the bottom left is the coronal view and the bottom right is the sagittal view. (a) 3D/3D matching of planCT to CBCT; (b) image scroll in axial view until the 2 off‐axis bbs displayed, then drag the green dash line up to where the side bb is (on the coronal view, the blue dash line is right on the side bb); (c) drag the blue dash line in coronal/sagittal view to planCT's isocenter (tiny green circles), then drag CBCT image up until the side bb is on the blue dash‐line; (d) with the red dash line set also passing through planCT's isocenter, in the axial view drag the CBCT image to bottom right until planCT's isocenter aligns with the two off‐axis bbs in CBCT.

Figures 5(a)–5(d) show the sequence of shifting planCT's isocenter to the off‐center point defined by the two off‐axis bbs in CBCT. They were captured from the 3D/3D matching window. In each of these figures, the top left is the axial view, the bottom left is the coronal view, and the bottom right is the sagital view. (The top right is the planCT view only and thus not shown here.) Figure 5(a) shows the 3D/3D matching of planCT to CBCT. After the matching, the couch vertical (Vrt), longitudinal (Lng) and lateral (Lat) shifts were 0.0, 0.1, and −0.1cm, respectively. In Fig. 5(b) we used the image scroll function in axial view until the two off‐axis bbs were displayed. We then dragged the green dash line up to the bb on the right side. On the coronal view, the blue dash line is right on the bb. In Fig. 5(c) we first dragged the blue dash line in coronal/sagital view to planCT's isocenter (the tiny green circles). We then dragged the CBCT image up until the side bb is on the blue dash line. The couch Vrt, Lng and Lat shifts were 0.0, 1.6 and −0.1cm, respectively. Note that 3D/3D manual matching can only be made by moving the CBCT image. In Figure 5(d), with the red dash line set so that it passes through planCT's isocenter, in the axial view we dragged the CBCT image to bottom right until the planCT's isocenter aligned with the two off‐axis bbs on CBCT. The matching generated expected couch shifts of 1.5, 1.6 and 1.5 cm. Then after the couch movement, the data table shown in Fig. 4(b) was also used to document the expected shifts, the initial and final couch positions, and the actual shifts. Again the accuracy of couch shifts (i.e. the |expected – actual|) should be within 2 mm.

## IV. DISCUSSION

Figure 3(b) shows that for Lat kV in a Varian's Ix‐21 machine, the imager isocenter was much more accurate in the S/I direction than in the A/P direction. Gravity‐caused imager sagging may be the major reason. For AP MV, the mean accuracy in S/I was also better than that in R/L. The mean accuracy in S/I was similar for both AP MV and Lat kV. In general, the image isocenter's accuracy is a combination of the accuracy of retractable arm's calibration, and the stability of the arm and gantry during the rotation. For the couch shift tolerance of 2 mm, we considered: 1) that the couch reading accuracy is approximately 1.4 mm (considering each 1 mm reading accuracy before and after the motion); and 2) the light field crosshair and laser alignment accuracy, which is also approximately 1.4 mm before and after the motion. Overall, 2 mm was set as the limit.

We noticed that the central bb in MV imaging is less easily visible. It becomes easier to identify the center of the central bb when: 1) it is set not to overlap with the couch's tennis grid, 2) the operator zooms the MV image by a large factor; and 3) the operator sets the display window level (−1350,−200). We also note that for OBI, 2D/2D is matched by moving the DRRs (i.e. reference) but not kV/MV images. On the other hand, 3D/3D is matched by moving the CBCT but not the reference (i.e. planCT). We suggest that Varian make this consistent in a future release of OBI.

This QA procedure is based on the phantom measurement. For real patients, it should be noted that the matching is not based on the high contrast bbs but rather on the anatomical structures using either the manual matching or specific algorithms (e.g. the mutual information). The accuracy of the matching also depends on the imaging techniques (kV, mAs), the matching area covered (local ROI or global), the treatment site, and even the patient size. Thus, additional errors may be involved in matching.

## V. CONCLUSIONS

In this paper, a positioning QA procedure for Varian's OBI, PI and CBCT system was developed. The suggested QA tests assure that the imaging isocenter is reliable and the couch movement is also reliable for the verification of patient positioning, using either 2D/2D or 3D/3D matching. The procedure is robust and easy to implement. We recommended that the QA for 2D/2D may be made on a daily or weekly basis, and that for 3D/3D, it may be made on a monthly basis.

## ACKNOWLEDGEMENTS

We appreciate the comments from the anonymous reviewers which helped make this manuscript ready for publication.
